# Notes and News

**Published:** 1895-09-28

**Authors:** 


### Notes and News.

(Collected and Communicated.)

The health of Mons. Pasteur is causing great anxiety to his friends. Although immediate danger is denied, very grave symptoms are admitted to be present.

The health of the French troops in Madagascar is reported to be deplorable. So rapidly has marsh fever spread in some cases that the medical men are questioning whether it is not contagious.

The recent increase of small-pox in the metropolis has brought a good deal of attention to bear on the sanitation of Salvation Army shelters. Numbers of local authorities are now endeavouring to bring these shelters under the provisions of the Lodging House Act. Numbers of cases of small-pox have been clearly traceable to them.

Some time since we drew attention to bank notes as a means of disseminating infection. Actual proof is difficult to procure in such cases, but that bank notes certainly absorb harmful matter has been proved by the experience of a bank clerk in Vienna quite recently. The man in question had to count a large number of notes, and, as is commonly done, in order to turn them over more speedily he wetted his fingers. He rapidly experienced pain on his lips, a tumour ensued, and in spite of an operation he succumbed from the effects of the poison absorbed.

On and after October 1st, the Royal College of Surgeons and Physicians have given notice that the following regulation will be in force in connection with the medical schools: A candidate referred in chemistry and physics will be required before being admitted to re-examination to produce a certificate that he has received further instruction in those subjects, to the satisfaction of his teacher, at an institution recognised for the purpose by the examining board, for a period of not less than three months subsequent to the date of his reference.

The International Institute of Sociology holds its second congress on the 30th inst. at Paris.

The French Hospital has been equally fortunate with other metropolitan institutions in benefiting from the gains of horse-racing. Mr. Lebaudy, whose horse won at the Auteuil races, has sent 25,000 francs to the hospital.

We have made inquiries as to the Durham Memorial Fund referred to by a correspondent last week, and find that since he wrote the whole sum of £2,000 has been promised, thus making the memorial a complete success within four months of its inauguration. The subscriptions were obtained without advertising or special publicity, and the result should serve as an object lesson to show what can be done by proper management, just as the Andrew Clark Memorial, with its hundreds of wasted pounds, so act as a warning in the other direction.

In recognition of the work done by the Seamen's Hospital Society at their branch hospital in the Royal Victoria and Albert Docks for the employés of the Dock Company, the London and India Docks Joint Committee have granted the site on which the branch stands to the society free of rent. Since the establishment of the branch hospital in 1890 a ground-rent of £50 per annum has been paid. In addition to the above concession the Joint Committee of the Dock Companies has allowed the society the use of a piece of land, about forty yards square, adjoining the hospital. This will be laid out as a garden and recreation ground.

A snowball collection of old postage stamps emanating from the United States for the purpose, it is stated, of getting a cripple into an institution for treatment is questioned by *Truth*, a million stamps being the price of admission. Most of our readers in England have, doubtlessly, like ourselves, been besieged in a like manner by collectors for some such object. We, like *Truth*, are very doubtful as to the possibility of an institution receiving old postage stamps in return for treatment, and our own inquiries have failed to bring any such institution to light. But we have been given the address of a dealer who will buy a million stamps for £2, but it would be far kinder to ask for 6d. from one's friends than to collect hundreds of postage stamps for a charitable object.

The Weber Prize, founded by Dr. H. Weber, of the U.S.A., in memory of Dr. E. A. Parkes, will be awarded every three years for the best essay upon some branch of the subject of tuberculosis, especially with reference to pulmonary consumption in man. The value is 750 dollars. The first award will be in 1897, and the subject selected by the adjudicators is "The Means, Prophylactic or Curative, deemed by the Author to have value in the Control of Tuberculosis, especial regard being had to their application to Human Tuberculosis." The choice of subject was made by Drs. J. Pollock, Thorne Thorne, and Professor S. Greenfield, nominated by the President of the Royal College of Physicians, and the merits of the essays will be decided by them also. The competition is open to medical men of all countries. The essays must be sent in to the Royal College of Physicians, London, and Dr. E. Liveing will reply to all questions on the subject.

